# Evidence for Activation of Toll-Like Receptor and Receptor for Advanced Glycation End Products in Preterm Birth

**DOI:** 10.1155/2010/490406

**Published:** 2010-11-28

**Authors:** Taketoshi Noguchi, Toshiyuki Sado, Katsuhiko Naruse, Hiroshi Shigetomi, Akira Onogi, Shoji Haruta, Ryuji Kawaguchi, Akira Nagai, Yasuhito Tanase, Shozo Yoshida, Takashi Kitanaka, Hidekazu Oi, Hiroshi Kobayashi

**Affiliations:** Department of Obstetrics and Gynecology, Nara Medical University, 840 Shijo-cho, Kashihara, Nara 634-8522, Japan

## Abstract

*Objective*. Individuals with inflammation have a myriad of pregnancy aberrations including increasing their preterm birth risk. Toll-like receptors (TLRs) and receptor for advanced glycation end products (RAGE) and their ligands were all found to play a key role in inflammation. In the present study, we reviewed TLR and RAGE expression, their ligands, and signaling in preterm birth. *Research Design and Methods*. A systematic search was performed in the electronic databases PubMed and ScienceDirect up to July 2010, combining the keywords “preterm birth,” “TLR”, “RAGE”, “danger signal”, “alarmin”, “genomewide,” “microarray,” and “proteomics” with specific expression profiles of genes and proteins. *Results*. This paper provides data on TLR and RAGE levels and critical downstream signaling events including NF-kappaB-dependent proinflammatory cytokine expression in preterm birth. About half of the genes and proteins specifically present in preterm birth have the properties of endogenous ligands “alarmin” for receptor activation. The interactions between the TLR-mediated acute inflammation and RAGE-mediated chronic inflammation have clear implications for preterm birth via the TLR and RAGE system, which may be acting collectively. *Conclusions*. TLR and RAGE expression and their ligands, signaling, and functional activation are increased in preterm birth and may contribute to the proinflammatory state.

## 1. Introduction

Preterm birth (delivery prior to 37 weeks gestation) occurs in around 10% of all deliveries and is the most significant problem encountered in obstetrics including neonatal morbidity and mortality [1]. This disorder is a complex cluster of problems associated with socioeconomic, sociodemographic, sociobehavioral, environmental, medical, biological, and genetic risk factors [2, 3]. Infection and inflammation are important etiological factors in the development of preterm birth, since nearly 30% of preterm deliveries are associated with intrauterine infection [1, 4]. Maternal infection (e.g., chorioamnionitis) is often followed by a systemic fetal inflammatory response characterized by elevated levels of proinflammatory cytokines in the fetal circulation [1, 5]. 

A comprehensive mapping of the proteome and microarray analysis was provided by several investigators [6–15]. Recent studies demonstrated associations between elevated levels of circulating proinflammatory cytokines, particularly interleukin (IL) 6, IL-1beta, and tumor necrosis factor alpha (TNF-alpha), and preterm birth [1, 5]. These inflammatory cytokines might link the pathology of uterine contraction, uterine cervical ripening, and preterm premature rupture of membrane (pPROM). 

The harmful effects of cytokines are mediated by specific receptors for inflammation. Toll-like receptors (TLRs) are the most extensively studied signaling receptors that participate in the initiation of inflammation [16]. Several researchers have pursued the association of TLRs and cytokines with preterm labor. Stimulation of TLRs with their ligands has been shown to induce proinflammatory cytokine release in uterine epithelial cells, fetal membranes and placenta [17–20]. Activation of the innate immune system via TLRs might be implicated in the pathogenesis of uterine contraction, uterine cervical ripening and pPROM in the process of preterm birth. Recent studies demonstrated that, besides TLRs, specific receptors can interact with other endogenous ligands generated by cell death and tissue injuries. However, there have been relatively few studies on such receptors and results have not been consistent [1]. Therefore, the precise molecular mechanisms by which cytokine expression cause preterm birth are not clear. In this paper, we have tried to summarize recent findings on TLRs, specific receptors, their ligands and their implications in preterm birth.

## 2. Materials and Methods

The present paper reviews the English language literature for biological, pathogenetic, and pathophysiological studies on preterm birth. We searched MEDLINE (PubMed) electronic databases for a 20-year period (1990–2010), combining the keywords “preterm birth”, “TLR”, “RAGE”, “danger signal”, “alarmin”, “genome-wide,” “microarray,” and “proteomics” with specific expression profiles of genes and proteins. Several recent studies are discussed in the context of pathogenesis of preterm birth. Additionally, references in each paper were searched to identify potentially missed studies for a 20-year period. Here, we discuss promising molecular candidates for preterm birth.

## 3. Factors Predictive of Preterm Birth

Recent advances in the application of various platforms have facilitated the process of discovery of novel biomarkers of preterm birth. These analyses include the DNA microarray experiment, principal component analysis, pathway analysis, signaling networks analysis, or proteomics analysis using matrix-assisted laser desorption ionization time-of-flight mass spectrometry (MALDI-TOF MS) techniques. Recent progress in the microarray, or proteomics-based technologies allows researchers to perform screening to detect differentially expressed genes and proteins in preterm birth subjects. Maternal serum and plasma, amniotic fluid, cervicovaginal fluid, urine, or placental trophoblasts, fetal membranes or cord blood have been used to develop markers for preterm birth.

### 3.1. Genes Differentially Expressed in Preterm Birth

The first aim of this study was to review molecular factors predictive of preterm birth by reviewing biochemical research identifying gene and protein expression profiles. Specific genes differentially expressed in preterm birth subjects are shown in Table 1. Many genes that participated in inflammation and immune system, transcription factors and signals, metabolism and cytokines, proteases, ion channel, hormone, extracellular matrix, and coagulation were overexpressed in patients with preterm birth [21–38]. Furthermore, we have reviewed genome-wide analytical methods and high-throughput computational tools to determine whether specific gene “signatures” can be identified among preterm birth subjects and controls. The “inflammatory signature” of molecular events seems to be associated with subsequent preterm birth. We named this specific gene “signature” “preterm birth signature”. 

A number of “preterm birth signature” genes involved in inflammation and immune system were specifically expressed, suggesting that excessive induction of the inflammatory response is a well-characterized cause of preterm birth. The expression of cytokines (notably interleukin 1beta [IL-1beta], IL-6, IL-8, and tumor necrosis factor alpha [TNF-alpha]) by either the maternal, fetal, or placental tissues has been demonstrated to upregulate the activity of a number of uterine and cervical factors (e.g., prostaglandins and their receptors and several proteases such as matrix metalloproteinases), leading to premature initiation and progression of the parturition process [22]. The analysis of “preterm birth signature”-dependent signaling network provides evidence for genes such as Nuclear Factor (NF)-kappaB, mitogen-activated protein kinase (MAPK), P38MAPK, Akt, and Early growth response (Egr)-1. 

The S100A5 gene is a member of the S100 family of proteins containing 2 EF-hand calcium-binding motifs. Some members are secreted from cells upon stimulation, exerting cytokine- and chemokine-like extracellular activities via the Receptor for Advanced Glycation End products, RAGE (see below) [24]. S100 proteins also activate NF-kappaB by inducing phosphorylation of IKKalpha/beta, leading to increased IkappaBalpha phosphorylation. NF-kappaB is a transcription factor family classically associated with inflammation. Premature activation of NF-kappaB through exogenous or endogenous stimuli might contribute to preterm birth [28].

### 3.2. Proteins Specifically Present in Preterm Birth

Uterine cervix is composed of extracellular matrix components such as collagen, elastin, proteoglycans, hyaluronan, and others. Cervical remodeling includes softening, ripening, dilatation, and repair. New insights propose that infection-induced premature cervical remodeling is distinct from the normal process [39–42]. The molecular mechanisms and pathways governing preterm and term cervical ripening are analogous, but distinctly heterogeneous and diverse [43]. 

Systemic or local inflammatory effects can influence cervical tissue remodeling [21, 44]. Morphological and biochemical changes are as follows: increased expression of matrix metalloproteinases and relaxin receptor, decline in collagen content, and loosening of the connective tissue structure [45–47]. Labor is associated with “decidual activation” with increased proteolysis and subsequent degradation of extracellular matrix [48, 49]. It has been reported that expression of laminin alpha 3 (LAMA3), fibronectin, and collagen IV mRNA was low during early gestation but increased dramatically at preterm birth [48, 50]. Several proteins and peptides were present specifically in preterm birth subjects. They were classified into several functional pathways that were involved with preterm labor/birth: extra cellular matrix, cell structure, protease, innate immunity, and transporter [51, 52]. Specific biomarkers relevant for preterm birth are shown in Table 2. The development of an assay to detect cervicovaginal oncofetal fibronectin 1 (FN1) can be helpful in selecting women at risk for preterm delivery [48, 53, 54]. This assay lies in the high negative predictive values of the tests for reducing preterm delivery risk. Furthermore, phosphorylated IGFBP-1 is clinically useful for prediction of preterm birth [7, 55]. 

Tissue remodeling in the uterine cervix depends on precise networks to fine tune the balance between proteases and inhibitors. The balance between plasminogen activator inhibitor-1 (PAI-1) and urokinase (uPA) and tissue-type plasminogen activator (tPA) is an important determinant of proteolytic activity at the maternal-fetal interface [48, 49]. Cathepsins (CTPs) are peptidases that have biological roles in degrading extracellular matrix, catabolism of intracellular proteins, and processing of prohormones [48]. CTSL2 is predominantly involved in the turnover of the extracellular matrix [56]. Cystatin C, a cysteine protease inhibitor, is involved in processes such as degradation of collagen and inflammatory processes [48, 57]. Cystatin is a candidate marker of inflammation. A human prothrombin fragment-2 (F2) inhibits the release of nitric oxide (NO), PGE2, and pro-inflammatory cytokines through suppression of expression of inducible NO synthase (iNOS) and cyclooxygenase (COX)-2 mRNA [58, 59]. F2 also suppresses the LPS-induced NF-kappaB activation [59]. These results suggest that F2 inhibits the inflammatory responses through suppression of NF-kappaB activation [59].

Key mediators of the innate immune system during pregnancy are the natural antimicrobial peptides, including secretory leukocyte peptidase inhibitor (SLPI), elafin and the defensins, may account for some of the antimicrobial activity of amniotic fluid [5, 7, 60]. The higher levels of defensins in cervicovaginal fluid had a greater risk of delivering before 32 weeks, demonstrating that midpregnancy human levels were more informative to preterm birth risk [7, 60, 61]. The S100 family members, including S100A8 (Calgranulin A), S100A9 (Calgranulin B), and S100A12 (Calgranulin C), are highly predictive of intrauterine inflammation and preterm birth [7, 60, 62].

## 4. Exogenous and Endogenous Ligands (Danger Signals, and Alarmins) in Preterm Birth

The purpose of the second aim of this study was to undertake a comprehensive review of exogenous and endogenous ligands, their receptors, and downstream signaling in preterm birth subjects. The innate immune system possesses pattern recognition receptors (PRRs) that recognize pathogen-associated molecular patterns (PAMPs) that are specific to microbes. These PRRs include Toll-like receptors (TLRs), nucleotide-binding domain leucine-rich repeat containing receptors (NLRs), RIG-I-like RNA helicases (RLHs) and C-type lectin receptors (CLRs) [63]. 

### 4.1. Toll-Like Receptor (TLR)

TLRs are evolutionarily preserved pattern-recognition receptor molecules that recognize the molecular patterns of pathogens [64]. TLR activation has been implicated in the regulation of the innate immune system and inflammation as well as the pathology of a number of inflammatory diseases including infectious diseases, tissue injury and damage, inflammatory bowel diseases, ischemia-reperfusion conditions, autoimmune and neurodegenerative diseases, and cancer [64–66]. TLRs were expressed on several cell types including monocytes, macrophages, and leukocytes [67]. They have been linked to the perpetuation of chronic inflammatory responses. Each TLR family member recognizes a specific pathogen component, upon activation, triggers a signaling cascade leading to cytokine production and adaptive immune response [64].

Interestingly, TLRs 1-10 are expressed in the female reproductive tract [68]. The TLR gene expression levels in endometrial tissues are high in the perimenstrual period and low in the periovulatory period [69]. The TLR2 expression is compatible in epithelial cells and stromal cells, while the TLR4 expression is higher in stromal cells. They are also present in the pregnant uterus, placenta and amniotic membranes. Also, TLR2 and TLR4 are widely reported to be present on trophoblast cells [70]. Activation of TLRs on trophoblast cells influences immune cell recruitment, cytokine secretion, and decidual responses to invading pathogens [71]. Among the TLRs, TLR1, TLR2, TLR4, and TLR6 may contribute to several pregnancy pathologies associated with preeclampsia, intrauterine growth restriction, and preterm labor/birth (see TLR Ligands section) [71–76]. 

Experimental evidence in animals demonstrates that TLR4 activation leads to the development of preterm birth [77]. While these important observations from animal model data suggest a role for TLRs in preterm birth, it remains unknown whether alterations in TLR pathway activation contribute to systemic or local inflammation in preterm birth subjects. The important studies implicating TLRs were derived using small sample size, and the association with respective TLR2/TLR4 ligands, downstream signaling, and functional activation remains to be properly addressed. We highlight recent data that assign a role to TLR ligands in the pathophysiology of preterm birth [71].

In addition, the NLR (also known as NOD-like receptors) family of intracellular sensors is a crucial component of the innate immune system [63]. The cytoplasmic pattern recognition receptors, NOD1 and NOD2, are important for detecting intracellular bacteria. NOD mRNA expression was upregulated following treatment of trophoblast cells with LPS. The NOD activation in trophoblasts triggers an inflammatory response [78]. In relation to TLRs, there is relatively little in the literature suggesting a role for NLRs and other receptors in pregnancy complications.

### 4.2. TLR Ligands

**Figure 1:**
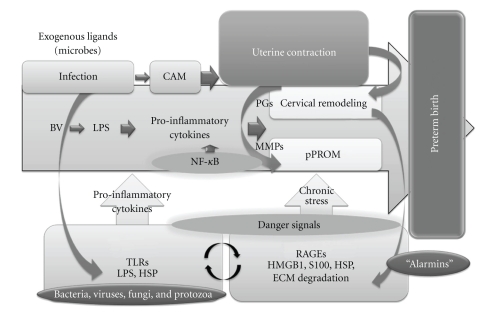
The inflammatory biomarkers, alarmins, involved in the development of preterm birth. The most frequently involved pathogens are thought to originate from the genital flora. Although TLR2 and TLR4 bind to components of the gram-positive and -negative bacteria, respectively, they recognize not only infectious agents (exogenous ligands) but also other endogenous ligands. Each TLR family member recognizes a specific pathogen component, upon activation, triggers a signaling cascade leading to pro-inflammatory cytokine production and adaptive immune response. Exogenous ligands for TLRs include bacteria, viruses, fungi, and protozoa as well as their components LPS, HSP and peptidoglycan. The downstream signaling network analysis provides evidence for genes such as NF-kappaB, MAPK, P38MAPK, Akt, and Egr-1. The expression of pro-inflammatory cytokines (notably IL-1beta, IL-6, IL-8, and TNF-alpha) by either the maternal, fetal, or placental tissues has been demonstrated to upregulate the activity of a number of uterine and cervical factors (e.g., prostaglandins and their receptors and several proteases such as matrix metalloproteinases), leading to premature initiation and progression of the parturition process. Activation of the pro-inflammatory and innate immune system via TLRs might be implicated in the pathogenesis of uterine contraction and pPROM in the process of preterm birth. The chronic inflammatory biomarkers, also known as “alarmins”, may be more important for the development of preterm birth. Intracellular “alarmins” are known as DAMPs, which includes HMGB1, HSPs, S100 proteins, and altered matrix proteins. “Alarmins” are secreted from cells upon stimulation, exerting cytokine- and chemokine-like extracellular activities via the RAGE. Increased expression of RAGE has been documented in preterm birth subjects. “Alarmins” might be more important for the development of preterm birth, leading to a chronic and persistent pro-inflammatory state by the activation of TLRs and RAGEs. TLR, Toll-like receptor; LPS, lipopolysaccharide; HSP, heat shock porotein; NF-kappaB, Nuclear Factor-kappaB; MAPK, mitogen-activated protein kinase; Egr-1, Early growth response-1; IL-1beta, interleukin-1beta; TNF-alpha, tumor necrosis factor-alpha; pPROM, preterm premature rupture of membrane; DAMPs, damage-associated molecular patterns; HMGB1, High-mobility group box 1; and RAGE, Receptor for advanced glycation end-products.

The most frequently involved pathogens are thought to originate from the genital flora (Gardnerella vaginalis, Mycoplasma hominis, Ureaplasma, Peptostreptococcus, Fusobacterium, Prevotella, and Bacteroides species) [79, 80] (Figure 1). Although TLR2, and TLR4 bind to components of the gram-positive and -negative bacteria, respectively [81], they recognize not only infectious agents (exogenous ligands) but also other endogenous ligands [71]. Ureaplasma species are commonly isolated pathogens from the female reproductive tract of women with preterm birth. Cell membrane lipoproteins from Ureaplasma can activate NF-kappaB through TLR1, TLR2, and TLR6 [76]. Thus, exogenous ligands for TLRs include LPS, lipoproteins, and peptidoglycan [72, 81]. 

#### 4.2.1. Lipopolysaccharide (LPS)

A number of animal studies have described a role of TLRs for controlling bacterial infection and its impact on preterm birth [82, 83]. Infection, including bacteria, viruses, fungi, and protozoa (e.g., sexually transmitted diseases or Gardnerella vaginalis), is thought to induce preterm birth through activation of inflammatory responses in both maternal and fetal tissues [82, 83]. This process initiates via signals through TLRs expressed by a wide spectrum of infectious microorganisms [83]. For example, the classic ligand that TLR4 recognizes is lipopolysaccharide (LPS) from gram-negative bacteria.

#### 4.2.2. Peptidoglycan

In placenta, gram-positive bacteria cell wall component peptidoglycan induces trophoblast apoptosis [84]. Furthermore, intraperitoneal peptidoglycan induced preterm delivery [84]. Peptidoglycan is a ligand of TLR2 [84]. Thus, activation of TLR2 can induce preterm delivery in mice.

### 4.3. Receptor for Advanced Glycation End-Products (RAGE)

Based on the proteomic discoveries, it has been propose that not only acute phase (IL-6 or PAMPs) but also chronic phase of the inflammatory and stress response is associated with preterm birth [7]. The chronic inflammatory biomarkers, also known as “alarmins”, may be more important for the development of preterm birth (Figure 1). Intracellular “alarmins” are known as damage-associated molecular patterns (DAMPs), which include HMGB1, HSPs, S100 proteins, and altered matrix proteins [7]. They represent important danger signals that mediate inflammatory responses through TLRs and importantly through RAGE. It is recognized that DAMPs mediate the late response to infection [7]. RAGE interacts with diverse ligands, including advanced glycation end products (AGEs), several members of the S100 protein family (S100B, S100P, S100A4, S100A6, S100A8/9, and S100A11–13), and high-mobility group box-1 (HMGB1) [85]. Increased expression of RAGE has been documented in a variety of acute and chronic inflammatory diseases [85].

### 4.4. RAGE Ligands

#### 4.4.1. Heat-Shock Protein 70 (HSP70)

Heat shock proteins (HSPs) such as HSP60, HSP 70, HSP72, and HSP 90 are representative endogenous ligands for TLRs or RAGE [86]. In general, HSPs are intracellular proteins with molecular chaperone and cytoprotective functions [73]. They play important roles in antigen presentation and activation of macrophages and lymphocytes [87]. HSP70 is present in the peripheral circulation of healthy nonpregnant and pregnant individuals. Elevated intracellular and extracellular HSP70 levels in healthy pregnant women at term might play a role in the onset of labor. Fukushima et al. reported that since HSP70 levels were particularly high in treatment-resistant preterm birth women, it may prove to be a useful marker for evaluating the effects of treatment or outcome [88]. 

Increased circulating HSP70 level may not only be a marker of these conditions but might also play a role in their pathogenesis [89]. Increased expression of HSP70 mRNA gene transcription has been observed in LPS-stimulated amniotic membranes [67]. Intra-amniotic infection, histologic chorioamnionitis, and term parturition are associated with elevated amniotic fluid HSP concentrations [67]. Extracellular HSP70 could engage with TLRs or RAGE to activate NF-kappaB and induce the production of pro-inflammatory cytokines including IL-1, IL-6, and TNF-alpha leading to prostaglandin production and preterm delivery [90, 91] (Figure 1). Also, HSP70 may stimulate the expression of prostaglandins and MMPs possibly through the TLR- and RAGE-mediated activation of COX-2 expression and result in the development of preterm labor and pPROM. The mechanisms of preterm and term parturition in humans may involve extracellular HSP70 [16]. Excess of the capacity of HSP70 to elicit the Th1-type immune responses might be harmful in pregnancy and leads to the rejection of the fetus.

#### 4.4.2. S100 Calcium-Binding Protein A4/A6

With great advancements in proteomics, new preterm birth biomarkers have been, and continue to be, discovered. Amniotic fluid biomarkers relevant for preterm birth are S100A12, S100A8, S100A9, defensin-1, defensin-2, and IGFBP-1 [7]. S100 proteins are ligands for the RAGE100A12 that has the strongest association with histological chorioamnionitis and funisitis [7]. Measurement of urine S100B protein levels in preterm newborns could be useful to identify newborns at higher risk of neonatal death [92].

#### 4.4.3. High-Mobility Group Box-1 (HMGB1)

Immune activation represents an adaptive reaction triggered by both exogenous (microbes) and endogenous inducers of inflammation [93]. High-mobility group box-1 protein (HMGB1), an evolutionarily conserved chromosomal protein, is one of the endogenous ligands [94]. HMGB1 was recently re-discovered to act as a “danger signal” (alarmin) to alert the innate immune system for the initiation of host defense or tissue repair [94]. Alarmin is a damage-associated molecular pattern molecule. Cell stress or necrosis leads the release of HMGB1 in the extracellular matrix, where it acts as an alarmin by engaging the RAGE [93]. HMGB1 levels correlated with levels of inflammatory markers, IL-6 and S100, in human fetus [93]. Animal model of LPS-induced preterm birth has demonstrated that inflammation induces a significant change in expression of RAGE and HMGB1 at sites of tissue damage [93]. These data suggest that RAGE and HMGB1 are important mediators of inflammation-induced preterm birth.

#### 4.4.4. Breakdown Products of Tissue Matrix—Extracellular Matrix Components

Proteins specifically present in preterm birth may have the properties of an endogenous alarmin. They include fibrinogen, fibronectin, heparan sulfate proteoglycan, hyaluronic acid, low-molecular weight hyaluronic acid, tenascin-C, neutrophil defensin 1, defensin 2, eosinophil-derived neurotoxin (EDN), lipocalin 2, fatty acid, apolipoprotein A-I, E and H (beta-2-glycoprotein I), oxidized LDL, lipoprotein, lipopeptides, annexin A2, amyloid beta A4 protein precursor, interferon- (IFN-) gamma, and lung surfactant protein, surfactant protein A (Table 2). Among them, endogenous ligands such as low-molecular weight hyaluronic acid, fibronectin, fibrinogen, HSP70, and heparin sulfate were found to be cleaved in the inflamed tissue and to activate their receptors.

Recent study on genetic polymorphism and contributions to disparities in preterm birth demonstrated that candidate genes include those involved in the host response to inflammation and those involved in the degradation of the extracellular matrix [95]. They include TNF-alpha, IL-1beta, IFN-gamma, and MMP-9. TNF-alpha is a pro-inflammatory cytokine that promotes an enhanced MMP (matrix metalloproteinases)/TIMP-1 (tissue inhibitors of metalloproteinases) ratio in the inflammatory states of preterm birth and points to potential mechanisms for cervical ripening and membrane rupture. Both TLRs- and AGEs/RAGE-dependent NF-kappaB signalings play key roles in TNF-alpha expression. These results suggest that the binding of “alarmins” to TLRs or RAGE activates various second-messenger systems including NF-kappaB, subsequently leading to the production of inflammatory cytokines such as TNF-alpha and MMPs. These findings may prompt new directions for targeting and treating preterm birth in future therapies.

## 5. Conclusions

This paper provides key evidence for increased TLR and RAGE expression, activation, exogenous and endogenous ligands, and downstream signaling contributing to inflammation seen in preterm birth subjects [74, 75, 88]. Associations have already been documented between TLR polymorphisms in man and TLR deficiency in animals and an increased susceptibility to infection and inflammation [96]. However, the functional state of the various components of RAGE and its ligands is largely unknown and only recently some studies have assessed this feature of the innate immune system. Therefore, the AGEs/RAGE system provides little critical insight into either the functional roles of the inflammatory signals or their downstream implications for preterm birth. 

The paper largely consists of a series of summaries reporting experimental or clinical evidence for the involvement of each particular receptor, factor, or downstream signaling system. “Alarmins” might be more important for the development of preterm birth, leading to a chronic pro-inflammatory state by the activation of TLRs and RAGE. RAGE has been implicated in chronic diseases such as diabetes, atherosclerosis, neurodisorders, cancers, and aging. Signalling pathways downstream of RAGE are activated by the accumulation of its ligands “alarmins.” A major event in the functional activation of TLRs and RAGE results in NF-kappaB activation and cytokine production [88]. RAGE binds a broad repertoire of ligands and may mediate responses to cell damage and stress conditions during preterm birth. A pro-inflammatory microenvironment is established by the secretion of cytokines, such as TNF-alpha, IL-1beta, and IL-6, and the production of RAGE ligands. Thus, RAGE ligands and subsequent signaling might stimulate uterine contraction, cervical ripening, and PROM by autocrine and paracrine feed-forward loops. 

In conclusion, the interactions between the TLR-mediated acute inflammation and RAGE-mediated chronic inflammation might have clear implications for preterm birth via the innate immune system. Taken together, the basic findings of this comprehensive review suggest that there is significant elevation of TLRs and RAGE, endogenous ligands, and cofactors in preterm birth patients, which contributes to the increase in chronic stress signaling and persistent pro-inflammatory state of preterm birth. These genes and proteins significantly elevated in preterm birth subjects may provide a foundation for further validation in larger patient cohorts. Future studies will address the mechanism of synergistic effects of endogenous ligands on TLR and RAGE signaling.

## Figures and Tables

**Table 1:** Genes differentially expressed in preterm birth.

Inflammation and immune system	IL-1beta (interleukin-1beta), IL-6, IL-8, TNF-alpha (tumor necrosis factor-alpha), S100A5 (S100 calcium-binding protein A5), P4HA2 (prolyl 4-hydroxylase, alpha polypeptide II), PTGDS (prostaglandin D2 synthase 21 kDa), VEGF (vascular endothelial growth factor), ABCB9 (ATP-binding cassette, subfamily B (MDR/TAP), member 9), and FCER1A (Fc fragment of IgE, high affinity I, receptor for; alpha polypeptide)

Transcription factor and signal	NF-kappaB (nuclear factor-kappaB), MAPK (mitogen-activated protein kinase), P38 MAPK, Akt, Egr-1 (early growth response-1), and HOX (homeobox)

Metabolism and cytokine	IL-1beta, TNF-alpha, ABP1 (amiloride-binding protein 1 or amine oxidase (copper-containing)), CBS (cystathionine-beta-synthase), SLC2 (solute carrier family 16, member 7 (monocarboxylic acid transporter 2)), and CCL2 chemokine (C-C motif) ligand 2 (MCP-1)

Protease	Ggt (gamma-glutamyl transpeptidase)

Ion channel	KCNH2 (potassium voltage-gated channel, subfamily H, member 2) and KCNMB4 (potassium large conductance calcium-activated channel, subfamily M, beta member 4, ion channel)

Hormone	Progesterone and Thyroid hormone

**Table 2:** Proteins specifically present in preterm birth.

Extra cellular matrix and cell structure	CLSTN1 (calsyntenin 1), DSP (desmoplakin), FN1 (fibronectin 1), IGFBP-1 (insulin-like-growth-factor-binding protein 1), LAMA3 (laminin alpha 3), LUM (lumican), and THSD1 (thrombospondin 1)
Protease	CTSL2 (cathepsin L2), CST (cystatin), PAI-1 (plasminogen activator inhibitor-1), TIMP1 (tissue inhibitor of metalloproteinase 1), uPA (urokinase plasminogen activator), F2 (prothrombin fragment 2), and SLPI (secretory leukocyte peptidase inhibitor)

Innate immunity	defensin-1, defensin-2, S100A8 (Calgranulin A), S100A9 (Calgranulin B), and S100A12 (Calgranulin C)

Transporter	Transthyretin (TTR)
